# Mask Optimization for High-Precision Extraction of Geometric Features in Microscopic Scenes

**DOI:** 10.3390/jimaging12060238

**Published:** 2026-05-28

**Authors:** Tianbo Kang, Jianpeng Zhang, Xin Zhao, Mingzhu Sun, Yunwang Zhang

**Affiliations:** 1 National Key Laboratory of Intelligent Tracking and Forecasting for Infectious Diseases, Engineering Research Center of Trusted Behavior Intelligence, Ministry of Education, Tianjin Key Laboratory of Intelligent Robotics, Institute of Robotics and Automatic Information System, Nankai University, Tianjin 300350, China; 1120240268@mail.nankai.edu.cn (T.K.); 2120240578@mail.nankai.edu.cn (J.Z.); zhaoxin@nankai.edu.cn (X.Z.); 2 Institute of Intelligence Technology and Robotic Systems, Shenzhen Research Institute of Nankai University, Shenzhen 518083, China; 3 Research Center of Laser Fusion, China Academy of Engineering Physics, Mianyang 621000, China

**Keywords:** microscopic scenes, regular geometric feature detection, object segmentation, iterative mask optimization

## Abstract

Regular geometric targets under microscopic scenes, such as microspheres, micropores, and microtubes, are characterized by small scales, low contrast, and degraded boundaries. Masks generated by general segmentation methods often fail to directly support high-precision geometric parameter measurement. This paper proposes a mask optimization method for the high-precision extraction of regular geometric features in microscopic scenes. We establish a mask optimization framework that integrates initial mask generation with geometric consistency refinement. Mask initialization is first performed through segmentation and adaptive super-resolution (SR) under low annotation constraints. Subsequently, an iterative optimization strategy that fuses multi-dimensional pixel features with regular geometric priors is designed. By incorporating geometric features extracted from the current mask while maintaining stable pixel-level observations, the mask is progressively corrected until convergence to generate target masks with continuous boundaries that satisfy stringent geometric constraints. Our experimental results on a sphere–tube assembly dataset demonstrate that the proposed method achieves lower geometric errors on successfully fitted samples and significantly improves the fitting success rate. Ablation studies further confirm the critical roles of dynamic SR and iterative mask optimization in enhancing overall precision and stability. These findings suggest that for microscopic regular geometric measurement tasks, integrating geometric-consistency constraints into mask optimization effectively improves both the accuracy and robustness of geometric feature extraction.

## 1. Introduction

Microscale assembly and precision manufacturing demand high-accuracy positioning and attitude measurement of microscale components. For regular geometric targets, such as microspheres, micropores, and microtubes, the geometric parameters (e.g., center position, radius or aperture, and central axis direction) serve as critical inputs for closed-loop control and quality assessment [1]. However, microscopic imaging is often degraded by the extremely small scales of the targets, low contrast, noise and blur, partial occlusion, or broken edges. Under such limited-data and complex imaging conditions, reliably extracting high-precision geometric features remains a challenging visual measurement problem.

Object detection or keypoint/parameter regression methods are often misaligned with the goals of high-precision geometric measurement. Mainstream detectors, such as the YOLO series, Faster R-CNN, and RetinaNet [2,3,4], are typically optimized for class prediction and bounding box localization, and they only provide coarse-grained geometric descriptions. They cannot impose precise constraints on object boundaries and fine shape details, and they are particularly prone to localization fluctuations and scale instability on tiny objects, which further affects the accuracy of subsequent geometric parameter estimation [5]. Traditional keypoint detection or direct regression of geometric parameters (e.g., circle center and radius) still fails to achieve global modeling of complete object boundaries. Such methods are sensitive to local observation deviations under noise, occlusion, and edge missing conditions, leading to limited model generalization and stability [6].

For regular geometric targets, traditional approaches typically extract discrete boundary points using edge detection operators and subsequently derive geometric parameters based on these points [7,8,9]. While this workflow yields accurate estimations under ideal imaging conditions, its fundamental limitation resides in its heavy dependency on clear and continuous edge responses, rendering it sensitive to threshold configurations, noise levels, and contrast variations. In microscopic scenes, factors such as boundary blurring, fragmented edges, and spurious edges introduce significant outliers among the discrete boundary points. Consequently, these outliers lead to instability in parameter estimation, elevated error margins, and even complete fitting failure [10,11].

Furthermore, traditional edge detection extracts only isolated local features and fails to generate holistic, continuous masks. This lack of global modeling capability for complex backgrounds and local defects makes it challenging to achieve a balance between robustness and high precision within a single framework. In contrast, pixel-level segmentation directly generates complete and continuous masks, forming dense and global boundary constraints at the contour level. This approach addresses the core limitations of the two aforementioned categories of methods, enabling subsequent geometric fitting to fully leverage the target’s global structural information. Consequently, it mitigates the interference of noise from discrete boundary points on fitting results, thereby enhancing the precision of parameter estimation [12,13]. Therefore, in microscopic measurement tasks for regular geometries, the strategy of fitting based on segmentation results possesses inherent advantages. Specifically, boundary errors introduced during segmentation are explicitly quantified and iteratively corrected at the mask level, while the subsequent fitting stage further incorporates global geometric constraints, significantly improving the stability and interpretability of the estimation [14].

However, the existing segmentation methods struggle to balance some core requirements for microscopic scenes, such as low annotation cost, strong cross-scene adaptability, and high-precision boundary representation. Traditional methods based on thresholding, edge detection, and morphological operations are sensitive to illumination fluctuations, low contrast, and noise, thereby lacking robustness under the complex degradations typical of microscopic imaging [15]. Learning-based methods are further classified into Generic Image Segmentation (GIS) and Promptable Image Segmentation (PIS) according to their inference-stage input paradigms and task objectives [16].

GIS models (e.g., U-Net, Mask R-CNN, and DeepLab [17,18,19]) rely on large-scale pixel-level annotated data for full supervision training, which is economically infeasible for rapidly changing industrial microscopic scenes [20]. In contrast, due to the pre-training on large-scale public datasets, the PIS method completes the segmentation only via corresponding prompts, which fully matches the engineering requirements of low annotation and high portability in industrial scenarios. Based on the prompt type, PIS can be subdivided into three categories. Interactive segmentation (e.g., the SAM series [21,22,23]) relies on manual tips, such as point and box, unable to meet the automated deployment of microscopic scenarios. Referring segmentation using language prompts leads to a lack of the fine-grained adaptability required for industrial micro targets. Few-shot segmentation requires targeted fine-tuning or domain adaptation, which has insufficient accuracy in cross-scene generalization [24,25].

Context-learning segmentation, serving as vision generalists (exemplified by the original Painter and SegGPT methods proposed in 2023 [26,27]), performs end-to-end inference using context prompt pairs of reference images with masks and query images, without additional training or fine-tuning. Requiring only minimal reference masks, this paradigm precisely fits the engineering deployment needs of microscopic measurement while offering superior cross-scenario adaptability. Therefore, this study adopts the context-learning approach as the core of the segmentation module, which provides a solid foundation for initial mask generation of microscopic targets under low-annotation constraints.

Although the targets can be segmented in the microscopic scenario, the scale of the targets changes significantly. For tiny objects with extremely low pixel occupancy, native models lack sufficient boundary representation capability, leading to issues such as boundary blurring, localization fluctuations, and incomplete contours. These defects are exponentially amplified during subsequent geometric fitting, directly degrading measurement accuracy. Current research often introduces super-resolution (SR) to enhance image details and improve segmentation accuracy. In the microscopic field, SR has been proven effective in improving boundary gradients and detail separability [28], but the existing methods face clear limitations. Global SR methods [29,30] upscale the entire image indiscriminately, introducing unnecessary computational overhead and potential background artifacts. Conversely, target-focused SR typically employs predefined, fixed enlargement factors [31], failing to dynamically adjust the strategy based on the actual target scale, thus struggling to balance computational efficiency with boundary enhancement gains. To this end, building upon context-learning segmentation, this paper introduces an adaptive SR strategy based on target scale estimation. This approach provides a reliable, boundary-enhanced initialization for subsequent mask optimization.

Even with boundary enhancement via adaptive SR, a fundamental mismatch persists between pre-trained segmentation foundation models and high-precision geometric measurement tasks. These models are primarily optimized for cross-domain generalization [32,33] rather than the boundary precision and shape-consistency constraints required for metrology. Consequently, the output masks still frequently exhibit defects such as edge shifts, local omissions, holes and noise. These defects are visually subtle and do not impair overall target integrity during segmentation. However, even small boundary deviations are greatly amplified in geometric fitting, resulting in an error explosion. This is the core challenge of microscopic high-precision geometric measurement, and also the key problem to be solved in this paper. To address this, we propose a mask-optimization method tailored for microscopic regular-geometry measurement. First, initial masks are obtained via segmentation and adaptive SR. Subsequently, an iterative optimization strategy that fuses multi-dimensional pixel features (intensity, gradient, edges, etc.) with regular geometric priors is designed to complete missing regions, remove noise, and enhance boundary consistency. The refined masks satisfy stringent geometric consistency requirements, providing a reliable foundation for subsequent geometric fitting and high-precision parameter estimation. This strategy effectively bridges the gap between general segmentation outputs and the rigorous demands of high-precision geometric detection, maintaining exceptional robustness under challenging conditions such as uneven illumination, partial occlusion, fragmented boundaries, and blurring.

To evaluate the efficacy of the proposed framework, a dedicated sphere–tube assembly dataset was curated for extensive experimental validation.

The main contributions of this paper include the following:

1. A two-stage mask optimization framework for high-precision measurement of microscopic regular-geometry targets is proposed. We establish a complete pipeline comprising adaptive SR enhanced initial mask generation and geometry prior-driven iterative mask optimization. This framework addresses the fundamental issue wherein masks output by general segmentation models cannot directly support high-precision geometric measurement, providing a high-robustness solution for microscopic metrology under low annotation cost.
2. An iterative mask optimization method is proposed by fusing multi-dimensional pixel features (e.g., intensity, gradient, and edges) with regular geometric priors, which rectifies boundary defects in the initial masks through explicit geometric constraints, substantially enhancing the precision and stability of geometric parameter estimation.
3. A dedicated dataset for microscopic regular geometries in sphere–tube assembly scenes is curated, covering a variety of typical microscopic degradation scenes. Through comparative and ablation experiments, the effectiveness, robustness, and engineering viability of the proposed mask optimization framework and its core modules were comprehensively validated.

## 2. Methods

Aiming at the high-precision measurement requirements for regular geometric targets in microscopic scenes, this paper proposes a two-stage mask generation and optimization framework. The framework is designed to generate high-quality target masks that stably support subsequent geometric fitting. The process is divided into two progressive core stages:

Stage I involves initial mask generation based on segmentation, combined with adaptive SR based on target scale to enhance boundary representation for small targets.

Stage II fuses multi-dimensional pixel features with regular geometric priors. It iteratively optimizes the masks while enforcing geometric-consistency constraints, ultimately outputting refined masks for high-precision geometric parameter estimation.

### 2.1. Stage I: Initial Mask Generation Based on SegGPT and Adaptive Super-Resolution

**Figure 1:**
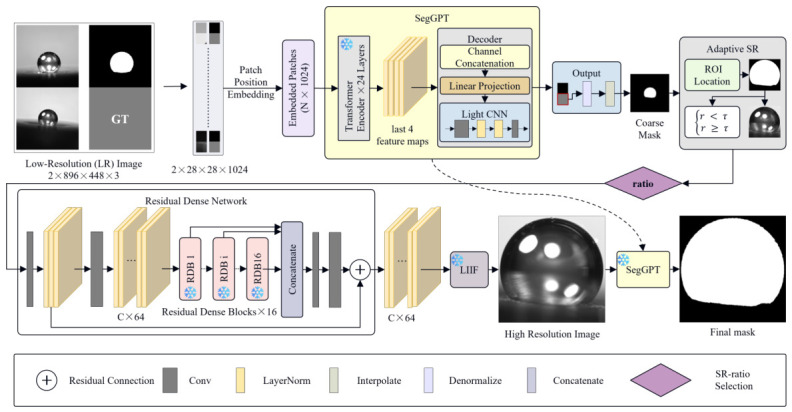
Stage I: Initial mask generation via SegGPT and Adaptive Super-Resolution.

Stage I aims to obtain a reliable initial mask under few annotation constraints. By employing target-scale adaptive SR, it enhances the boundary quality of small-scale targets, providing a stable initialization for the subsequent geometry-driven optimization. The complete workflow of Stage I, as shown in Figure 1, consists of three steps: (1) initial segmentation based on context learning; (2) target scale estimation and SR factor decision; and (3) refined mask generation after target-scale adaptive SR enhancement.

#### 2.1.1. Segmentation Based on Context Learning

To reduce pixel-level annotation reliance and improve transferability across regular geometric targets, we utilize the model SegGPT [27] for segmentation. Specifically, for each target class, a reference pair $\left(I_{LR},M_{LR}\right)$ is constructed, where $I_{LR}\in R^{H\times W\times 3}$ is the reference image and $M_{LR}$ is the corresponding reference mask, which explicitly represents the target’s shape prior (e.g., spherical, elongated tubular, or dual-contour pore). During the inference stage, the reference pair and the target image $I_{t}$ are concatenated according to 1:1, resized to the model’s required input scale (e.g., 448×448), and divided into patches with positional encoding. Global context features are extracted through a multi-layer pre-trained Transformer encoder [34]. To reduce complexity and enhance boundary response, multi-scale features are fused via a lightweight convolutional network in the decoding stage, generating a binary coarse mask:

(1) $$\hat{M_{0}}\in {\left\{0,1\right\}}^{H\times W},$$

Binarization and lightweight morphological opening are applied to suppress isolated noise, ensuring the mask provides robust coverage of the target in local regions.

#### 2.1.2. Scale Estimation and Adaptive SR Factor Decision

Target sizes vary greatly in microscopic scenes, and details depend heavily on resolution. Small-target segmentation thus often has unsmooth boundaries, missing parts, or noise. We estimate the target scale based on the initial mask $\hat{M_{0}}$ and adaptively select an SR magnification factor to balance computational cost and detail enhancement. The Region of Interest (ROI) is derived from the minimum bounding box of $\hat{M_{0}}$, expanded by δ pixels to mitigate boundary truncation errors. The ROI scale index *S* is calculated as:

(2) $$S=\frac{w\times h}{W\times H},$$

where W×H is the original image size and w×h is the ROI size. A scale ratio $k=S/S^{*}$ is defined by comparing *S* with a predefined target scale $S^{*}$, which determines the SR factor r∈1,2,4,…. When *k* is low, a higher SR factor is applied to enhance boundaries and fine structures; otherwise, a lower factor (or no SR) is used to avoid redundant computation and the introduction of artifacts. This adaptive strategy effectively compensates for size differences between the input image and the target resolution at different ratios, ensuring consistent boundary representation quality for targets of different scales.

#### 2.1.3. SR Enhancement and Refined Mask Generation

After determining *r*, SR reconstruction based on the Local Implicit Image Function (LIIF) is performed on the ROI to obtain the enhanced image $I_{R}$. Unlike fixed-kernel or fixed-ratio methods, LIIF learns a continuous implicit representation, mapping discrete low-resolution images to color/intensity functions in a continuous coordinate domain. This allows for arbitrary magnification (e.g., 2× to 12×), producing smoother grayscale transitions at edges. After obtaining $I_{R}$, the same strategy is applied to $I_{R}$ to generate the refined mask $M_{0}$. Due to the enhanced boundary gradients and improved local texture discriminability provided by SR, $M_{0}$ typically outperforms $\hat{M_{0}}$ in contour continuity and detail completeness, making it more suitable for subsequent geometry-constrained optimization.

### 2.2. Stage II: Iterative Mask Optimization with Multi-Dimensional Features and Geometric Priors

**Figure 2:**
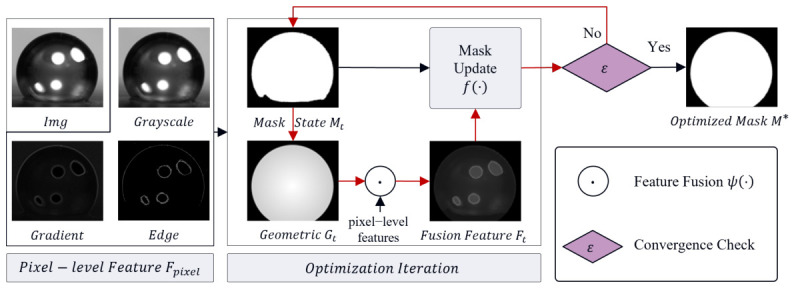
Stage II: iterative mask optimization with multi-dimensional features and geometric priors. * denotes the optimal mask state.

Building upon $M_{0}$, Stage II models mask optimization as a closed-loop iterative process. By progressively introducing regular geometric priors, the initial segmentation is subjected to stable and controllable consistency refinement. Unlike one-step post-processing, this stage treats the mask as a state variable that is dynamically adjusted until it converges to a result satisfying geometric constraints. The workflow is illustrated in Figure 2 and detailed in Algorithm 1.

The pixel-level feature extractor Φ(I) is a fixed heuristic function with no learnable parameters. It generates a 3-channel feature map $F_{pixel}\in R^{H\times W\times 3}$ from the original input image:

(3) $$F_{pixel}=\left[I_{norm},\nabla I,E_{adap}\right],$$

where $I_{norm}$ is the normalized grayscale image scaled to [0, 1], ∇I is the gradient magnitude map, and $E_{adap}$ is the binary edge map. All channels share the same spatial dimension as *I* and remain constant throughout the optimization process to provide stable local observations.

The geometric feature extractor $\phi (M_{t})$ generates a dense single-channel Signed Distance Field (SDF) map $G_{t}\in R^{H\times W}$ from the current mask $M_{t}$. In each iteration, we first extract all foreground pixel coordinates $P=\{\left(x_{i},y_{i}\right)|M_{t}\left(x_{i},y_{i}\right)=1\}$ and fit the target parametric geometric model Θ from *P* using least squares. For circular targets (microspheres, micropores), $\Theta =(x_{c},y_{c},r)$ representing center coordinates and radius; for linear targets (microtube axes), Θ=(a,b,c) representing the line equation ax+by+c=0. Then, we compute the SDF map via pixel-wise broadcasting:

(4) $$G_{t}\left(x,y\right)=d\left(\left(x,y\right),\Theta \right),$$

where d(·) is the signed distance from pixel (x,y) to the ideal geometric boundary defined by Θ, with negative values indicating pixels inside the target and positive values indicating pixels outside.
**Algorithm 1** Mask optimization based on multi-dimensional features and geometric priors**Require:**      1.Original image $I_{LR}\in R^{H\times W\times 3}$      2.Initial mask $M_{0}$ (from Stage I)      3.Maximum iterations $T_{max}$      4.Convergence threshold ε**Ensure:** Optimized final mask $M^{*}$
 1:Extract pixel-level features (intensity, gradient, edges) from $I_{LR}$: 2:   $F_{pixel}=\Phi \left(I\right)$ 3:Initialize state variables: 4:   Set iteration counter t←0, initial mask state $M_{t}\leftarrow M_{0}$. 5:**while** $t<T_{max}$ **do** 6:     Update geometric features: calculate features based on current mask $M_{t}$: 7:        $G_{t}=\phi \left(M_{t}\right)$ 8:     Multi-dimensional fusion: fuse pixel-level and geometric features: 9:        $F_{t}=\psi \left(F_{pixel},G_{t}\right)$10:     Update mask: update state based on $F_{t}$ and $M_{t}$:11:        $M_{t+1}=f\left(F_{t},M_{t}\right)$12:     Convergence check:13:     **if** $∥M_{t+1}-M_{t}∥<\varepsilon$ **then**14:            **break**15:     **else**16:            t←t+1, $M_{t}\leftarrow M_{t+1}$17:     **end if**18:**end while**19:Output final state: $M^{*}=M_{t}$.


As shown in Figure 2 and detailed in Algorithm 1, the algorithm first extracts fundamental pixel-level features (grayscale, gradient, edges) fused into a unified representation $F_{pixel}$. This feature remains constant during optimization to provide stable local observations. Then, using $M_{0}$ from Stage I as the initial state, the process enters a closed-loop refinement. In each iteration, the geometric feature $G_{t}$ is calculated from $M_{t}$ to characterize the target’s global geometric attributes. Then, $G_{t}$ is fused with $F_{pixel}$ via the function ψ(·) to obtain the comprehensive feature $F_{t}$:

(5) $$F_{t}=\psi \left(F_{pixel},G_{t}\right),$$

where ψ(·) is explicitly defined as a weighted element-wise addition with fixed weights $\omega_{p}=0.9$ and $\omega_{g}=0.1$ for all experiments:

(6) $$F_{t}=\omega_{p}\cdot F_{pixel}+\omega_{g}\cdot G_{t},$$

This representation encodes both local pixel evidence and global geometric consistency. On this basis, the current mask is gradually refined through the mask update function to generate a new mask state $M_{t+1}$.

The mask update function $f(F_{t},M_{t})$ is a non-parametric heuristic operation without learnable parameters. Given the adaptive threshold T=μ+0.2σ (where μ and σ denote the mean and standard deviation of $F_{t}$) the mask is updated by an asymmetric dual-direction rule:

(7) $$M_{t+1}\left(p\right)=\left\{\begin{array}{cc} 1, & \mathrm{if}\;M_{t}\left(p\right)=1\;\mathrm{and}\;F_{t}\left(p\right)\geq 0.8T\\ 1, & \mathrm{if}\;M_{t}\left(p\right)=0\;\mathrm{and}\;F_{t}\left(p\right)\geq 1.1T\\ 0, & \mathrm{otherwise} \end{array}\right.$$

A 3 × 3 morphological closing operation is applied to refine mask connectivity, and a foreground protection constraint is enforced to retain target integrity.

To ensure the stability and convergence of the optimization process, the algorithm determines whether the convergence condition is met by the change in the mask after each round of iteration:

(8) $$\|M_{t+1}-M_{t}\|<\varepsilon ,$$

where ‖·‖ denotes the Frobenius norm, which measures the difference metrics, such as pixel-wise error or IoU change. When the difference falls below ε, the mask shape is considered stable. The final mask $M^{*}$ significantly improves upon the initial result in terms of boundary continuity and geometric consistency, enabling high-precision geometric fitting.

## 3. Experiments and Results

### 3.1. Experimental Platform and Dataset

To evaluate the performance of the proposed method in high-precision detection tasks for regular geometric targets, validation experiments were conducted on a self-built sphere–tube assembly dataset containing microspheres, micropores, and microtubes. All our experiments utilized consistent data partitioning, the same geometric feature-fitting algorithms, and unified evaluation metrics to ensure comparability across the different methods. The specific scale of the dataset is detailed in Table 1.

**Figure 3:**
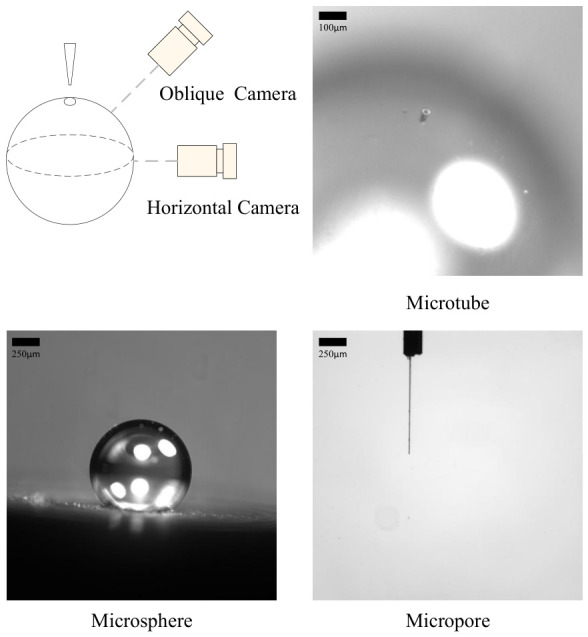
Microscopic imaging system and dataset example: the **upper left** shows a schematic diagram of the multi-channel microscopic imaging system for the tube assembly scene; the **upper right** is the microscopic image of the micropore target captured by the tilting camera; the **lower left** is the microscopic image of the microsphere target captured by the horizontal camera; the **lower right** is a microscopic image of the microtube target captured by a horizontal camera.

The imaging system and representative examples of the three typical targets in the dataset are illustrated in Figure 3. The image resolutions covered two typical settings: 2448 × 2050 and 5120 × 5120. The dataset comprised both clear images and complex scenes characterized by varying degrees of noise, blur, occlusion, and local defects.

For the three typical targets covered by the aforementioned sphere–tube assembly dataset, the core task of this paper was the high-precision geometric parameter detection of regular geometry targets in microscopic scenes. Key pose parameters of microspheres, micropores, and microtubes were extracted from microscopic images to provide high-precision measurement inputs for the closed-loop control of the assembly process, including, specifically, center coordinate detection for microspheres, center coordinate detection for micropores, and axis position and orientation detection for microtubes. These corresponded to the core engineering requirements of microsphere localization, micropore posture adjustment, and microtube posture adjustment during the assembly process.

To quantitatively evaluate the detection accuracy and methodology performance, considering the distinct geometric features of the three targets, we adopted sphere center positioning error, pore center positioning error, and microtube axis angle error as the primary evaluation metrics. The ground-truth masks and geometric parameters were manually annotated using the VGG Image Annotator (VIA) tool in high-magnification view, with cross-checks to ensure pixel-level annotation precision for reliable evaluation.

To avoid statistical interference caused by fitting failures or severely abnormal results, the average error metrics were computed exclusively on samples where geometric fitting was successfully completed. In addition to the geometric error metrics, we also report the fitting success rate to comprehensively evaluate the robustness of the different methods. A sample was considered successfully fitted if the estimated geometric parameters were within reasonable bounds: center error < 1000 px for microspheres, <100 px for micropores, and angle error < 10° for microtubes. The fitting success rate was calculated on the complete test set without any sample exclusion, while the average error was only calculated on successfully fitted samples to ensure the validity of the error statistics.

### 3.2. Comparative Experiment

Traditional segmentation methods, such as thresholding and edge detection, often struggle with the complex degradation typical of microscopic imaging, including non-uniform illumination and low contrast. Since these methods fail to provide the stable, closed mask structures required for high-precision geometric fitting, they did not serve as competitive benchmarks for this task.

Learning-based methods are categorized as GIS and PIS models. This study excluded standard GIS models from direct comparison because they rely on large-scale, domain-specific labeled datasets to achieve high accuracy. Such heavy data dependency contradicts the low-annotation requirements of microscopic industrial scenes.

Consequently, this study focused on a performance comparison with several representative PIS foundation models, as they represent the state-of-the-art in flexible and robust target localization. All the methods utilized the same geometric feature-fitting pipeline and parameter settings to ensure that the results accurately reflected how the quality of segmentation masks affects the final geometric detection performance.

**Figure 4:**
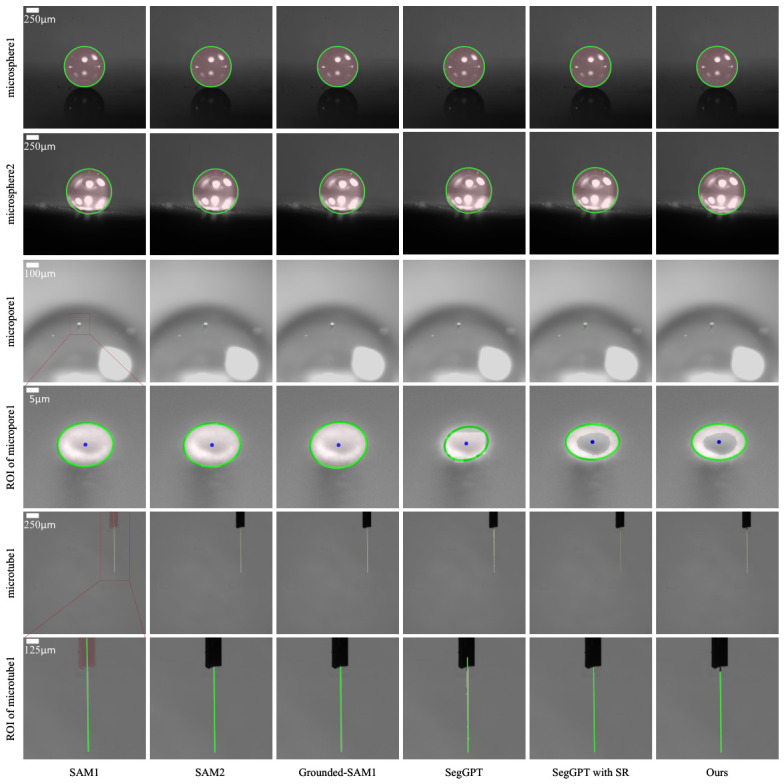
Segmentation and geometric fitting effects of different methods on microsphere, micropore and microtube samples. Green lines represent the fitted geometric results: circular and elliptical contours for microspheres and micropores, and the central axis for microtubes.

The segmentation masks generated by the different methods and their corresponding geometric fitting results are illustrated in Figure 4. It can be observed that the mainstream PIS foundation models successfully extracted visually complete target regions in most samples. However, their mask boundaries still exhibited local defects, which affected the subsequent geometric fitting accuracy. In contrast, the masks generated by the proposed method were more consistent with the prior characteristics of regular geometric targets in terms of structural continuity and boundary consistency. This effectively suppressed fitting errors and enhanced the precision and stability of the parameter estimation, providing a more robust and reliable input for the subsequent geometric fitting.

To comprehensively evaluate the performance of different methods from both robustness and accuracy perspectives, we first report the fitting success rates of all the methods on the complete test set, as shown in Table 2.

The geometric feature-fitting performance on successfully fitted samples is presented in Table 3, where the reported values denote the average geometric fitting errors computed over valid samples. Across the three categories of targets, the proposed method achieved lower average geometric errors than the general-purpose segmentation methods, with a particularly pronounced advantage in microtube angle estimation. These results indicate that while general segmentation methods possess strong panoptic segmentation capabilities, their outputs lack consistency constraints tailored for regular geometric structures, making it difficult to directly satisfy the requirements of high-precision metrology. All statistical comparisons were performed using the Wilcoxon signed-rank test to verify the significance of performance differences between the methods.

In terms of computational efficiency, the proposed mask optimization module achieved an average of 3.2 iterations across all the test samples, and 99% of all the samples converge within 5 iterations without any divergence. The overall inference speed of the complete framework was also evaluated on a single NVIDIA RTX 4060 GPU (Nvidia Corporation, Santa Clara, CA, USA), with an average runtime of approximately 4 s per testing image with 5120 × 5120 resolution, which is sufficient for most industrial offline and online microscopic measurement tasks.

**Figure 5:**
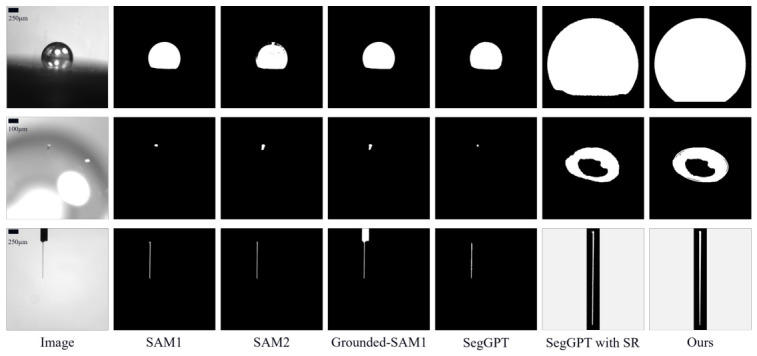
Comparison of segmentation masks for different methods.

A further analysis of the results where the comparative segmentation methods yielded significant fitting errors or failures revealed a high incidence of fitting failures and abnormal errors in certain samples, as illustrated in Figure 5. These are primarily attributable to unclosed mask structures or a lack of local geometric consistency. By contrast, the proposed method achieved a significantly higher fitting success rate across all three categories of targets, demonstrating its superiority in terms of geometric consistency and practical utility.

**Figure 6:**
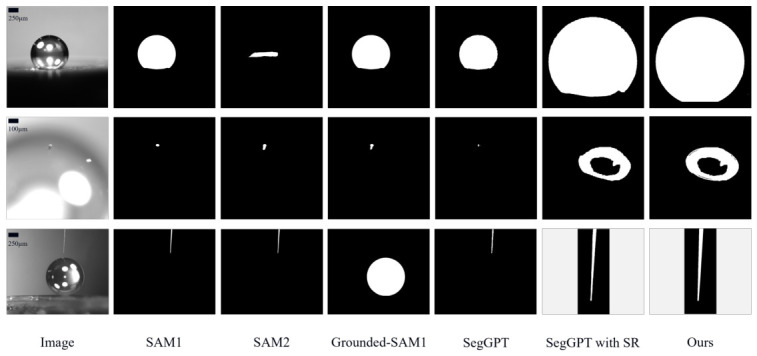
Representative failure cases of mainstream segmentation methods and the corresponding robust results obtained by our method.

Figure 6 further illustrates representative failure cases of mainstream segmentation methods. These methods frequently generated fragmented or distorted masks, whereas our method yielded complete and geometrically consistent results.

### 3.3. Ablation Study

Building upon the overall performance advantages demonstrated in the comparative experiments, ablation studies were further conducted to analyze the specific contributions of each key module to the geometric detection performance. Using the basic segmentation results of SegGPT as the baseline, we sequentially integrated the adaptive SR processing and the iterative mask optimization modules to compare their performance across the three types of targets. The mask visualization results for each module are presented in Figure 5, labeled as SegGPT, SegGPT with SR, and Ours, and the corresponding fitting effects are illustrated in Figure 4.

In the baseline segmentation results of SegGPT, although basically usable target regions were obtained for some samples, the mask boundaries commonly exhibited discontinuities or local offsets, which limited the stability of geometric fitting. With the introduction of adaptive SR, edge details and local structural representations were enhanced, improving mask continuity and generally reducing geometric fitting errors. However, when the initial mask contained significant structural defects, relying solely on resolution enhancement was insufficient to fully recover a shape that satisfied the geometric constraints. By further incorporating iterative mask optimization, the mask was progressively corrected under regular geometric consistency constraints, making its overall morphology more reflective of the target’s true geometric structure. This significantly enhanced both the stability and precision of subsequent fitting.

The performance of these variations is summarized in Table 4, where the reported values denote the average geometric fitting errors computed over fitted samples. It can be observed that adaptive SR consistently reduced errors for micropores and microtubes. However, for microspheres, the sphere center error for SegGPT with SR increased slightly compared to the original SegGPT. Analysis shows that this anomaly occurred because the resolution enhancement also magnified tiny impurities and stains near the microsphere edges. Since circle-fitting algorithms typically assume continuous, noise-free edge points they become more sensitive to such outliers at higher resolutions. Notably, mask iterative optimization further reduced the average geometric error and significantly decreased the number of failed or abnormal samples, validating the robustness and effectiveness of the proposed framework in complex microscopic scenes.

In summary, adaptive SR and iterative mask optimization not only improve geometric detection accuracy within our framework but also significantly enhance the overall stability and usability of the method in complex microscopic environments.

To further validate the necessity of the proposed adaptive SR strategy, a controlled experiment was conducted by replacing the super-resolution module with SwinIR [35]. To ensure a fair evaluation of the SR component’s contribution, the entire downstream pipeline, including SegGPT segmentation, mask optimization, and geometric fitting, was kept strictly identical, with only the SR module modified.

Unlike LIIF, which natively supports arbitrary continuous magnification and is therefore configured with adaptive scaling factors, SwinIR is pre-trained for standard discrete scaling factors. Prior research shows that standard SwinIR requires 4.88 s per image on an NVIDIA RTX 4090 (NVIDIA Corporation, Santa Clara, CA, USA), even at moderate scales [36], and that it is 4.34× slower than efficient CNN counterparts on NVIDIA V100 GPUs (NVIDIA Corporation, Santa Clara, CA, USA) [37]. Therefore, we restricted SwinIR to a fixed 2× magnification to comply with industrial latency constraints. This configuration represents a pragmatic compromise between geometric fidelity and throughput in resource-constrained microscopic inspection systems.

The geometric accuracy of the SwinIR-based seg+sr+opt pipeline is summarized in Table 5. In this controlled setting, the fixed 2× SwinIR configuration exhibited a mean pore-center deviation of 18.93±11.23 px.

This systematic accuracy degradation is primarily attributable to the resolution and magnification mismatch between the compared SR configurations. The adaptive LIIF framework dynamically selected optimal magnification factors based on each ROI’s native resolution, thereby maximizing boundary sampling density for subsequent ellipse fitting. In contrast, the fixed 2× magnification inevitably reduced boundary-pixel sampling during the secondary SegGPT segmentation and propagated coarser geometric estimates through the ellipse-fitting stage. This result quantifies the fundamental precision–latency trade-off inherent to microscale geometric metrology. Higher SR magnification improves downstream fitting stability but increases computational burden. Consequently, the more lightweight computational profile of LIIF enables adaptive 3–4× magnification to remain feasible for industrial deployment, whereas the heavier SwinIR architecture necessitates a lower fixed magnification, directly impacting downstream geometric precision. These findings validate the design rationale of the proposed adaptive SR strategy, demonstrating that dynamic magnification selection is critical for balancing real-time constraints with metrology-grade accuracy in microscopic regular-geometry measurement.

## 4. Discussion

This study proposes a high-precision microscopic geometric feature extraction framework that combines adaptive SR with iterative mask optimization. Our experimental results demonstrate that the proposed method achieves an order-of-magnitude improvement in geometric parameter extraction for microspheres, micropores, and microtubes compared with mainstream PIS foundation models. The core reason for this performance gap lies in the fundamental difference between the objective functions of the two approaches. The general segmentation model takes the integrity of the target mask region as the main optimization objective, and its output focuses on the integrity of the region rather than the mathematical accuracy of the boundary.

Combined with the introduction and further analysis of the experimental results, it can be found that general foundation models exhibit some inherent limitations for microscopic metrology. First, they lack geometric consistency, focusing solely on semantic segmentation while ignoring the inherent geometric constraints of regular targets. Second, they suffer from a subpixel precision bottleneck, where encoder downsampling loses high-frequency details, failing to deliver metrology-level boundary accuracy. Third, they have inherent output randomness, making results non-reproducible for precision measurement. Fourth, they lack industrial robustness, performing poorly under uneven illumination, surface impurities, and other industrial degradations. The proposed framework addresses these core limitations: global geometric prior optimization enforces geometric consistency; adaptive-scale super-resolution ensures uniform resolution for subpixel-level fitting; a fully deterministic pipeline guarantees reproducibility; and geometric constraints filter industrial interference to enhance robustness.

The results of the ablation study demonstrate that while adaptive SR processing may enhance the local continuity of object edges, it cannot recover shapes that satisfy geometric constraints when the initial mask contains significant structural defects or noise. In such cases, if subsequent optimization is lacking then SR may instead amplify noise and artifacts, resulting in an increase in fitting errors. The mask iterative optimization module functions precisely on this basis. It treats the mask as an updatable state variable and performs closed-loop correction by fusing pixel features with geometric rules. This effectively compensates for fragmented boundaries and suppresses random jitter, serving as a critical link in achieving high stability.

This mechanism is particularly evident in the microsphere task, where the error increase of SegGPT with fixed SR is mainly caused by the over-enhancement of local surface protrusions, which leads to obvious bulges in the initial mask and further degrades fitting accuracy. The proposed method, through the global constraint of circular geometric prior, can automatically eliminate such local abnormal structures and naturally tolerate normal structural occlusions common in industrial scenarios, restoring the ideal geometric parameters of microspheres without any additional preprocessing. On this basis, the maximum positive residual score may be used to automatically detect surface protrusions. For a fitted circle with center $\left(x_{c},y_{c}\right)$ and radius *r*, the maximum positive residual is defined as:

(9) $$\Delta_{\max}=\max\left(0,\max_{i=1..N}\left(\sqrt{{\left(x_{i}-x_{c}\right)}^{2}+{\left(y_{i}-y_{c}\right)}^{2}}-r\right)\right)$$

where $\left(x_{i},y_{i}\right)$ are the coordinates of the *i*-th contour point, and where *N* is the total number of contour points. Samples with $\Delta_{\max}$ exceeding a predefined threshold are marked as having surface defects. This mechanism requires no additional computation and can provide value-added functions for industrial quality inspection while ensuring measurement accuracy.

In the automated assembly process for microscale precision components, such as the sphere–tube assembly, high-precision geometric localization is a prerequisite for closed-loop robotic control. The tens-of-pixels error produced by general models cannot meet the requirements for micron-level alignment and may even lead to equipment damage. The performance of the method proposed in this paper on the tube assembly dataset proves that the framework possesses the potential for direct integration into industrial automation systems. Meanwhile, the adaptive SR ensures precision while avoiding global computation, providing feasibility for deployment in real-time industrial vision systems. Furthermore, the proposed iterative mask optimization module is a completely independent and training-free post-processing component. It may seamlessly integrate with the output of any existing segmentation network (such as SAM and SAM2) without any modification, which significantly improves the generalizability and practical value of our method.

It should be emphasized that this work is specifically targeted at parametric regular geometric targets in industrial microscopic assembly scenarios, which account for the vast majority of industrial micro-assembly components. The proposed method can be easily extended to other regular geometric shapes by simply replacing the corresponding geometric fitting function without modifying the core optimization modules. In practical industrial deployment, our method is robust to domain shift across microscopes, requiring only one reference sample for adaptation. It has been stably deployed on multiple devices with consistent accuracy.

In addition, it should be noted that the current method still relies on a multi-stage pipeline, which carries a certain risk of error propagation. Furthermore, the geometric constraints are primarily oriented toward specific regular shapes. The applicability to more geometric shapes remains to be validated. Future work will explore end-to-end geometric parameter regression frameworks and further evaluate the generalization capability across multiple devices and various lighting conditions.

## 5. Conclusions

Aiming at the pivotal challenge of high-precision detection of regular geometric targets in microscopic assembly, this paper proposed a mask optimization framework integrating segmentation, adaptive SR, and regular geometric priors. Centered on the core goal of enhancing the suitability of masks for precision metrology, the multi-stage framework was developed to overcome challenges such as small target scales, significant boundary degradation, and the inadequacy of general segmentation results for high-precision metrology.

The framework acquires initial masks by context learning and enhances local boundary representation through an adaptive SR strategy. By further introducing an iterative optimization mechanism that fuses pixel features with geometric priors, the resulting masks significantly outperform general segmentation results in terms of boundary continuity and global geometric consistency. Our experimental results on self-built datasets of microspheres, micropores, and microtubes demonstrate that the proposed method consistently achieved a center positioning error of 0.75 px and an axial angle error of 0.01° under various imaging qualities and complex interferences. These results validate the high precision and stability of the framework, ensuring robust engineering applicability for high-precision autonomous assembly and measurement in microscopic scenes.

In summary, this work indicates that in microscopic regular geometric measurement tasks, combining general-purpose segmentation models with explicit geometric-consistency constraints to perform targeted mask optimization is an effective approach for enhancing both detection accuracy and robustness. Nevertheless, the current method relies on a multi-stage pipeline and targets specific regular geometries. Future work will focus on end-to-end regression and broader geometric generalization.

## Figures and Tables

**Table 1:** Data description of the sphere–tube assembly dataset.

Target Type	Quantity (Images)
Microsphere images	416
Micropore images	474
Microtube images	385

**Table 2:** Fitting success rates of different methods on microsphere, micropore, and microtube samples.

Method	Microsphere (%)	Micropore (%)	Microtube (%)
SegGPT	100.0	99.6	100.0
SegGPT with SR	100.0	100.0	100.0
SAM	100.0	100.0	100.0
SAM2	96.6	99.6	100.0
Grounded-SAM	100.0	69.4	99.0
Ours	100.0	100.0	100.0

**Table 3:** Average geometric fitting errors of different methods on microsphere, micropore, and microtube samples.

Method	Sphere Center Error (px)	Pore Center Error (px)	Microtube Angle Error (°)
SAM	32.43 ± 20.30 **	22.34 ± 23.49 **	0.46 ± 0.43 **
SAM2	30.42 ± 20.32 **	62.48 ± 21.38 **	0.39 ± 0.33 **
Grounded-SAM	29.46 ± 19.51 **	38.13 ± 19.45 **	0.44 ± 0.47 **
Ours	0.75 ± 0.28	0.75 ± 0.29	0.01 ± 0.01

Note: All values are presented as mean ± standard deviation; ** denotes statistical significance (p<0.001) compared with the proposed method.

**Table 4:** Average geometric fitting errors of ablation experiment on microsphere, micropore, and microtube samples.

Method	Sphere Center Error (px)	Pore Center Error (px)	Microtube Angle Error (°)
SegGPT	22.51 ± 13.57 **	15.57 ± 10.46 **	0.44 ± 0.35 **
SegGPT with SR	27.96 ± 18.78 **	0.73 ± 0.30	0.39 ± 0.33 **
Ours	0.75 ± 0.28	0.75 ± 0.29	0.01 ± 0.01

Note: All values are presented as mean ± standard deviation; ** denotes statistical significance (p<0.001) compared with the proposed method.

**Table 5:** Geometric accuracy of the SwinIR-based pipeline on micropore samples.

Method	Target	Metric	Mean ± Std (px)
seg+sr+opt (SwinIR 2×)	Micropore	Pore Center Error	18.93 ± 11.23

## Data Availability

The data presented in this study are available on request from the corresponding author due to privacy restrictions.
